# The Effects of Anlotinib Combined with Chemotherapy following Progression on Cyclin-Dependent Kinase 4/6 Inhibitor in Hormone Receptor-Positive Metastatic Breast Cancer

**DOI:** 10.1155/2024/5396107

**Published:** 2024-08-02

**Authors:** Ting Xu, Weili Xiong, Lili Zhang, Yuan Yuan

**Affiliations:** ^1^ Department of Oncology The Affiliated Cancer Hospital of Nanjing Medical University, Nanjing, China; ^2^ Department of Chemotherapy Jiangsu Cancer Hospital Jiangsu Institute of Cancer Research The Affiliated Cancer Hospital of Nanjing Medical University, Nanjing, China

## Abstract

**Purpose:**

Endocrine therapy combined with cyclin-dependent kinase (CDK) 4/6 inhibitors (CDK4/6i) is the preferred treatment for hormone receptor-positive (HR+)/human epidermal growth factor receptor 2-negative (HER2–) metastatic breast cancer (MBC). However, there are currently no recommendations for therapeutic strategies after progression on CDK4/6i-based treatment. This study aimed to examine the efficacy and safety of anlotinib plus chemotherapy in HR+/HER2– MBC after progression on CDK4/6 inhibitors.

**Methods:**

We collected data from 32 patients with HR+/HER2– MBC treated with anlotinib plus chemotherapy after progressing on CDK4/6i at Jiangsu Cancer Hospital from March 2020 to October 2023. The median follow-up was 9.1 months (range, 2.0–19.7 months) as of the data cutoff date in October 2023. The primary endpoint was median progression-free survival (PFS); secondary endpoints included objective response rate (ORR), disease control rate (DCR), and adverse events.

**Results:**

The median PFS (mPFS) of all patients was 7.6 months (95% confidence interval (CI), 5.75–9.45). There was no significant difference in mPFS between patients who responded to prior CDK4/6i treatment and those who did not (8.3 months vs. 6.8 months, *p*=0.580). Besides, the ORR was 34.4% and DCR was 93.8%. The most frequently observed adverse events were anemia (50.0%), neutropenia (40.6%), thrombocytopenia (34.4%), and epistaxis (34.4%). Dose interruption or reductions due to adverse events occurred in 2 (6.3%) and 5 (15.6%) patients, respectively.

**Conclusions:**

The study preliminarily demonstrates that anlotinib combined with chemotherapy may be an optional recommendation for patients with HR+/HER2– metastatic breast cancer who have progressed after CDK4/6i.

## 1. Introduction

Breast cancer (BC) is the most common malignancy among women [1], of which approximately 65%–70% are hormone receptor-positive (HR+)/human epidermal growth factor receptor 2-negative (HER2–) diseases [2]. For these patients, cyclin-dependent kinase (CDK) 4/6 inhibitor plus endocrine therapy has become the preferred treatment, tremendously improving progression-free survival (PFS) and even demonstrating benefits in overall survival (OS) [3–5]. However, almost all patients would eventually develop disease progression, necessitating a transition to different therapeutic approaches. Currently, there are no recommendations for therapeutic strategies after progression on CDK4/6i combined with endocrine treatment, posing urgent needs for clinicians in China to address this issue.

Several clinical studies, both domestically and abroad, have explored various approaches after progression on CDK4/6i. These include switching to another CDK4/6i [6], combining endocrine therapy with other targeted agents like alpelisib [7, 8], everolimus [9, 10], and tucidinostat [11], as well as antibody-drug conjugate (ADC), such as sacituzumab govitecan [12] and trastuzumab deruxtecan [13], or chemotherapy [14]. Unfortunately, despite the positive outcomes observed in the BYLieve trial, which demonstrated alpelisib's effectiveness after progression on CDK4/6i-based treatment, it is not approved in China. Besides, the mutation rate of PIK3CA is very low [8], making it unsuitable for the majority of patients. Other strategies have generally fallen short of achieving satisfactory efficacy, with a median progression-free survival (PFS) ranging between 2 and 6 months. Given the limitations in accessibility and efficacy, there is an urgent need for new treatment regimens.

Anlotinib is a novel multitargeting tyrosine kinase inhibitor that targets vascular endothelial growth factor receptor (VEGFR), fibroblast growth factor receptor (FGFR), platelet-derived growth factor receptors (PDGFR), and c-kit [15]. In our previous study, anlotinib-based treatment demonstrated effectiveness in the multiline treatment of MBC patients who failed the standard treatment [16].

Currently, there are no clinical data on the efficacy of anlotinib in combination with chemotherapy in HR+/HER2– MBC with progression on prior CDK4/6i-based treatments. The primary objective of this study was to analyze the efficacy and safety of anlotinib combined with chemotherapy in patients whose disease progressed after CDK4/6i treatment.

## 2. Materials and Methods

### 2.1. Data Source and Study Population

This single-institution, retrospective study received approval from the ethics board of Jiangsu Cancer Hospital (No. 2020 − 042), and individual consent for this retrospective analysis was waived.

All female patients with HR+/HER2– MBC who underwent treatment with anlotinib combined with chemotherapy after progressing on the CDK4/6i treatment between Mar 1, 2020, and Oct 11, 2023, at Jiangsu Cancer Hospital were included. We collected medical data from 32 patients who received at least two-cycle anlotinib combined with chemotherapy treatments after CDK4/6i treatment.

### 2.2. Procedures

Patients were treated with anlotinib combined with chemotherapy, primarily utilizing anti-microtubule drugs such as taxanes, eribulin, and utidelone. Nab-paclitaxel was administered at 125 mg/m^2^, paclitaxel at 80 mg/m^2^, and eribulin at 1.4 mg/m^2^ on days 1 and 8, every 21 days per cycle. Utidelone was given at 30 mg/m^2^ for 5 days, every 21 days per cycle. The initial dose of anlotinib varied, with patients receiving 12 mg, 10 mg, or 8 mg, respectively, as determined by the clinician. Anlotinib was administered orally once daily continuously for 2 weeks, followed by one week off, completing a 21-day cycle. Dose adjustments, permanent discontinuation, or delays were allowed based on patients' side effects [17, 18].

Following palliative chemotherapy, patients received maintenance therapy with anlotinib in combination with endocrine therapy until disease progression, unacceptable toxicity, or death.

### 2.3. Assessment

The primary endpoint of this study was progression-free survival (PFS), which was defined as the duration between anlotinib-based treatment, including chemotherapy and endocrine therapy after progression on CDK4/6i regimen, and the date of disease progression or death. Other assessments of the therapeutic effects included the objective response rate (ORR) and disease control rate (DCR). ORR was defined as the proportion of patients achieving a complete response (CR) or partial response (PR) to therapy while DCR included patients with CR, PR, or stable disease (SD) in response to therapy.

Sensitivity to CDK4/6i combined with endocrine therapy was defined as a documented clinical benefit equal to or more than 6 months from the initiation of CDK4/6i treatment [11, 14].

Treatment response was routinely assessed at baseline and every two cycles with chemotherapy and every three cycles with endocrine as maintenance treatment by computed tomography (CT) or magnetic resonance imaging (MRI), based on the Response Evaluation Criteria in Solid Tumors (RECIST 1.1). Adverse events were evaluated according to the Common Terminology Criteria for Adverse Events version 5.0 (CTCAE 5.0).

### 2.4. Statistical Analysis

Statistical analyses were conducted using SPSS version 26.0 (IBM, New York, USA). The Kaplan–Meier curves were employed for analyzing PFS, and stratified log-rank tests were utilized to obtain *P* values between groups. Additionally, the Cox proportional hazards model was employed to calculate hazard ratios (HRs) and corresponding 95% confidence intervals (CIs). *P* value <0.05 was considered indicative of statistical significance.

## 3. Results

### 3.1. Patient Characteristics

A total of 32 patients, with a median age of 53 years (range, 36–73), were enrolled in this study, and their baseline characteristics are summarized in Table 1. All enrolled patients had previously undergone various CDK4/6i treatments and endocrine therapy. Among them, 81.3% of patients had visceral metastasis. Additionally, 56.3% of the patients were sensitive to the prior CDK4/6i treatment, and 50.0% of patients received anlotinib in combination with chemotherapy sequentially (immediately after disease progression on the initial CDK4/6i). However, the remaining 50% of patients received other treatments prior to anlotinib. Among them, most patients received chemotherapy and continued endocrine maintenance therapy after the chemotherapy regimens until disease progression.

The median number of anlotinib with chemotherapy treatment lines was 4 (range, 2–6), and the most common chemotherapy partner of anlotinib was taxanes (37.5%), followed by eribulin (25.0%), utidelone (15.6%), and others (21.9%).

### 3.2. Efficacy

The median follow-up was 9.1 months (range, 2.0–19.7 months) as of the data cutoff date in October 2023. At that point, seven patients (21.9%) were still under treatment, while twenty-four patients (75.0%) had discontinued treatment due to disease progression, and one patient (3.1%) was lost to follow-up.

**Figure 1:**
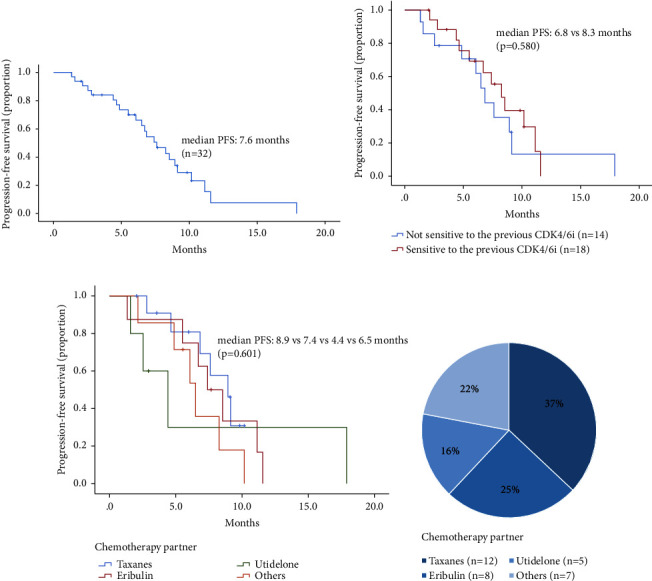
Kaplan–Meier curves were generated for median progression-free survival (mPFS) analysis in the following groups: (a) all patients, (b) patients stratified by sensitivity to the previous CDK4/6i treatment, and (c) patients with different chemotherapy partners of anlotinib. (d) The percentage of patients receiving various chemotherapy partners of anlotinib after prior CDK4/6i progression. “Others” include capecitabine, vinorelbine, and epirubicin.

The median PFS was 7.6 months (range, 1.3–17.9) (Figure 1(a)), with a six-month progression-free survival rate of 56.3%. When comparing patients sensitive and not sensitive to CDK4/6 inhibitors, there was no statistically significant difference in efficacy, although the sensitive group had a longer mPFS (8.3 months, 95% CI: 6.43–10.11, vs. 6.8 months, 95% CI: 5.61–8.05, *p* = 0.580 (Figure 1(b)).

Stratified by different chemotherapy partners of anlotinib, the mPFS was 8.9, 7.4, 4.4, and 6.5 months for taxanes, eribulin, utidelone, and others (including capecitabine (*N* = 3), vinorelbine (*N* = 2), and epirubicin (*N* = 2)), respectively (*p*=0.601 (Figures 1(c) and 1(d))). Furthermore, the median PFS was 6.8 months (95% CI, 5.35–8.32 months) in patients who received sequential anlotinib plus chemotherapy treatment after CDK4/6i progression, which was shorter than that of those who received non-sequential treatment ((8.3 months, 95% CI, 5.15–11.39, *p*=0.250)).

**Figure 2:**
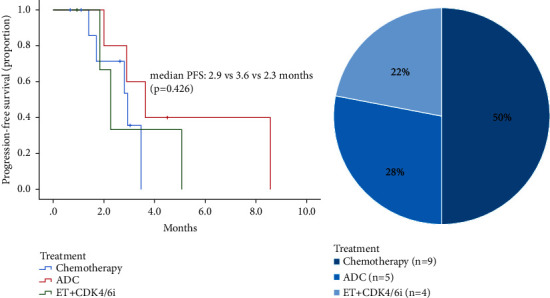
(a) Kaplan–Meier curves of mPFS for patients stratified by different treatment regimens after anlotinib combined with chemotherapy progression. (b) The percentage of patients receiving different treatment regimens after anlotinib combined with chemotherapy progression.

Among the 24 patients who discontinued anlotinib combined with chemotherapy due to disease progression, 6 patients were lost to follow-up. The remaining 18 patients were followed up with different treatment regimens, including chemotherapy (50.0%), antibody-drug conjugate (28.0%), and CDK4/6i combined with endocrine therapy (22.0%). In detail, the chemotherapy regimens consisted of taxanes (*N* = 2), eribulin (*N* = 3), and utidelone (*N* = 4). Antibody-drug conjugate refers to disitamab vedotin (*N* = 5), and CDK4/6i included dalpiciclib (*N* = 2) and palbociclib (*N* = 2). The median progression-free survival (mPFS) was 2.9, 3.6, and 2.3 months, respectively (*p*=0.426, Figure 2).

**Figure 3:**
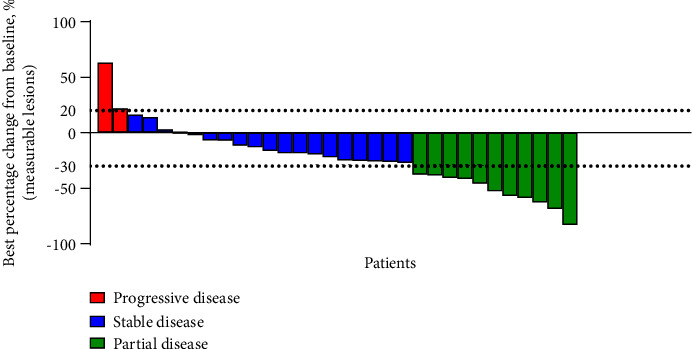
A waterfall plot illustrating the best percentage change in the sum of the diameters of target lesions at any time in patients with measurable disease at baseline who received anlotinib combined with chemotherapy treatment.

The analysis of the best overall response involved 32 patients. None of the patients achieved complete response (CR), 11 achieved partial response (PR), 19 had stable disease (SD), and 2 developed progressive disease (PD), resulting in an overall response rate (ORR) of 34.4% and disease control rate (DCR) of 93.8% (Figure 3). In addition, stratified by different chemotherapy partners of anlotinib, the ORR was 25.0%, 37.5%, 20.0%, and 57.1% for taxanes, eribulin, utidelone, and others, and the DCR was 100.0%, 87.5%, 80.0%, and 100.0%, respectively.

In this study, univariate and multivariate analysis showed that no factors were significantly related to PFS in log-rank analysis (Table 2).

### 3.3. Safety

Adverse events in all patients are detailed in Table 3. There were no treatment-related deaths or instances of drug discontinuation due to adverse effects recorded in the study. Adverse reactions resulted in treatment interruption for 2 patients (6.3%) and dosage reductions for 5 patients (15.6%). The most common adverse event was anemia (50.0%), followed by neutropenia (40.6%), thrombocytopenia (34.4%), and epistaxis (34.4%). The most frequent grade 3 or 4 treatment-related adverse events were neutropenia (21.9%). Additionally, increased aspartate/alanine aminotransferase (31.3%), diarrhea (31.3%), oral mucositis (21.9%), and hypertension (21.9%) were also observed. However, most patients deemed these reactions tolerable, and symptomatic treatments provided relief.

## 4. Discussion

Endocrine therapy combined with CDK4/6 inhibitors has become the preferred treatment for HR-positive metastatic breast cancer. However, there is currently no optimal recommendation for patients with HR+/HER2– MBC who experience progression on CDK4/6 inhibitors. This study retrospectively analyzed the efficacy and safety of anlotinib combined with chemotherapy in patients with HR+/HER2– MBC who progressed on prior CDK4/6 inhibitor therapy for the first time, and the mPFS was 7.6 months (95% CI, 5.75–9.45 months).

There are some studies to explore the treatment strategy after the failure of CDK4/6i. After the failure of palbociclib, the mPFS of chemotherapy monotherapy remains at 4.1–5.6 months [14, 19]. In addition to chemotherapy, other target drugs, such as mTOR inhibitors, HDAC inhibitors, other CDK4/6 inhibitors, and PIK3CA inhibitors combined with endocrine therapy, were alternative options after CDK4/6 inhibitor progression. The efficiency was not satisfactory, with mPFS around 5 months. The mPFS of everolimus plus endocrine therapy was 3.6–4.2 months, and the mPFS of tucidinostat after CDK4/6 inhibitor progression in real-world studies varied from 2 to 5.1 months [9–11]. The replacement of ribociclib combined with endocrine therapy showed an mPFS of 5.29 months after CDK4/6 inhibitor progression [6]. These data confirmed the available measures were significantly shorter than anlotinib in combination with chemotherapy in China. The present study further analyzed the mPFS according to the chemotherapy agent. Anlotinib combined with taxanes seemed to show better efficacy, although the difference was not statistically significant (*p*=0.601).

For patients with PIK3CA mutations, the mPFS of alpelisib plus fulvestrant was 7.3 months [7, 8]. Unfortunately, alpelisib has not been approved in China, and the incidence of PIK3CA mutations seems low, observed in about 40% [8], making the drug unavailable for most patients. In our study, anlotinib combined with chemotherapy showed good efficacy, with mPFS benefits similar to alpelisib. Although lacking head-to-head comparative trials, it could be a preferred option after CDK4/6 inhibitor failure in China. Additionally, contrary to previous studies suggesting worse prognosis for patients not sensitive to CDK4/6 inhibitors [12], our study found no impact on the efficacy of subsequent anlotinib-based treatment, regardless of sensitivity.

Furthermore, we assessed mPFS based on whether anlotinib plus chemotherapy was used immediately after CDK4/6 inhibitor therapy progression. No significant difference in mPFS was observed, suggesting that the efficacy of anlotinib may not be affected by the number of therapy regimens between CDK4/6 inhibitors and anlotinib plus chemotherapy.

In our study, the most common adverse event was anemia (50.0%), with no grade 3 or 4 anemia observed. Although diarrhea was common, most patients found it tolerable. Bone marrow suppression, represented by neutropenia (21.9%), was the most frequent grade 3 or 4 treatment-related adverse event, but neutrophil counts usually returned to normal after recombinant human granulocyte colony-stimulating factor (rhG-CSF) treatment. Some patients experienced bleeding events, such as epistaxis and hemoptysis, associated with the increased risk of VEGFR-TKI-induced bleeding incidence [20]. However, bleeding symptoms were relatively mild and did not require intervention. In summary, the combination of anlotinib and chemotherapy was well tolerated, with no treatment-related deaths, and adverse events were generally manageable with current guidance.

This study has a few limitations, including being a single-center retrospective study with a small sample size. Due to the relatively short follow-up period, we cannot prove whether anlotinib combined with chemotherapy can affect the overall survival rate of MBC patients. Therefore, more prospective studies are needed to confirm these results in the future.

## 5. Conclusion

Our study suggests that anlotinib combined with chemotherapy may be an optional strategy for patients with HR+/HER2– metastatic breast cancer that has progressed on CDK4/6 inhibitors. However, its efficacy needs further confirmation through prospective studies with larger sample sizes.

## Figures and Tables

**Table 1:** Baseline patient characteristics.

Characteristics	Number of patients (%) (*n* = 32)
Age (years), median (range)	53 (36–73)
Menopausal status	
Premenopausal	9 (28.1%)
Postmenopausal	23 (71.9%)
Visceral metastases	
Yes	26 (81.3%)
No	6 (18.7%)
Dominant metastatic sites	
Liver	19 (59.4%)
Lung	18 (56.3%)
Bone	22 (68.8%)
Brain	7 (21.9%)
Lymph nodes	24 (75.0%)
Number of metastatic sites	
<3	5 (15.6%)
≥3	27 (84.4%)
CDK4/6 inhibitor	
Palbociclib	13 (40.6%)
Abemaciclib	8 (25.0%)
Dalpiciclib	5 (15.6%)
Other^a^	6 (18.8%)
Sensitive to the previous CDK4/6i	
Yes	18 (56.3%)
No	14 (43.7%)
Lines of therapy of anlotinib plus chemotherapy	
2	8 (25.0%)
3	3 (9.4%)
≥4	21 (65.6%)
Number of therapy regimens between CDK4/6i and anlotinib	
0	16 (50.0%)
≥1	16 (50.0%)
The therapy regimens between CDK4/6i and anlotinib	
Taxanes	11 (34.4%)
Eribulin	5 (15.6%)
Capecitabine	6 (18.8%)
Tucidinostat plus exemestane	5 (15.6%)
Chemotherapy partner of anlotinib	
Taxanes	12 (37.5%)
Eribulin	8 (25.0%)
Utidelone	5 (15.6%)
Others	7 (21.9%)

^a^Other refers to FCN-437c (*N* = 6), a novel CDK4/6i in clinical trials.

**Table 2:** Univariate and multivariate Cox regression analysis of factors affecting PFS.

Factors	Univariate analysis		Multivariate analysis	
	Hazard ratio (95% CI)	*P* value	Hazard ratio (95% CI)	*P* value
Age (>50 vs. ≤50)	0.58 (0.24–1.41)	0.231	0.70 (0.19–2.69)	0.608
Menopausal status (postmenopausal vs. premenopausal)	0.58 (0.24–1.41)	0.229	0.45 (0.16–1.30)	0.139
Visceral metastases (yes vs. no)	1.47 (0.49–4.48)	0.494	1.82 (0.35–9.34)	0.473
Number of metastatic sites (≥3 vs. <3)	2.79 (0.90–8.63)	0.075	2.24 (0.36–13.88)	0.385
Sensitivity to the previous CDK4/6i (yes vs. no)	0.79 (0.34–1.84)	0.581	0.80 (0.29–2.21)	0.673
Lines of therapy of anlotinib plus chemotherapy (>3 vs. ≤3)	1.96 (0.77–5.00)	0.158	2.20 (0.35–13.92)	0.404
Number of therapy regimens between CDK4/6i (≥1 vs. 0)	0.58 (0.23–1.48)	0.255	1.19 (0.24–5.88)	0.829

**Table 3:** Adverse events.

Adverse events	All grades (%)	Grade 3/4 (%)
Anemia	16 (50.0%)	1 (3.1%)
Neutropenia	13 (40.6%)	7 (21.9%)
Thrombocytopenia	11 (34.4%)	1 (3.1%)
Epistaxis	11 (34.4%)	0
Aspartate aminotransferase increased	10 (31.3%)	0
Alanine aminotransferase increased	10 (31.3%)	0
Diarrhea	10 (31.3%)	0
Oral mucositis	7 (21.9%)	2 (6.3%)
Hypertension	7 (21.9%)	0
Hand-foot syndrome	6 (18.8%)	0
Vomit	4 (12.5%)	0
Fatigue	3 (9.4%)	0
Rash	2 (6.3%)	0
Hemoptysis	2 (6.3%)	0
Blood bilirubin increased	1 (3.1%)	0
Adverse events leading to treatment interruption		2 (6.3%)
Adverse events leading to dose reduction		5 (15.6%)

## Data Availability

Original data are available upon reasonable request to the corresponding author.

## References

[1] Sung H., Ferlay J., Siegel R. L. (2021). Global cancer statistics 2020: GLOBOCAN estimates of incidence and mortality worldwide for 36 cancers in 185 countries. *CA: A Cancer Journal for Clinicians*.

[2] Eggersmann T. K., Degenhardt T., Gluz O., Wuerstlein R., Harbeck N. (2019). CDK4/6 inhibitors expand the therapeutic options in breast cancer: palbociclib, ribociclib and abemaciclib. *BioDrugs*.

[3] Finn R. S., Martin M., Rugo H. S. (2016). Palbociclib and letrozole in advanced breast cancer. *New England Journal of Medicine*.

[4] Johnston S., Martin M., Di Leo A. (2019). MONARCH 3 final PFS: a randomized study of abemaciclib as initial therapy for advanced breast cancer. *NPJ Breast Cancer*.

[5] Hortobagyi G. N., Stemmer S. M., Burris H. A. (2018). Updated results from MONALEESA-2, a phase III trial of first-line ribociclib plus letrozole versus placebo plus letrozole in hormone receptor-positive, HER2-negative advanced breast cancer. *Annals of Oncology*.

[6] Kalinsky K., Accordino M. K., Chiuzan C. (2023). Randomized phase II trial of endocrine therapy with or without ribociclib after progression on cyclin-dependent kinase 4/6 inhibition in hormone receptor-positive, human epidermal growth factor receptor 2-negative metastatic breast cancer: maintain trial. *Journal of Clinical Oncology*.

[7] Rugo H. S., Lerebours F., Ciruelos E. (2021). Alpelisib plus fulvestrant in PIK3CA-mutated, hormone receptor-positive advanced breast cancer after a CDK4/6 inhibitor (BYLieve): one cohort of a phase 2, multicentre, open-label, non-comparative study. *The Lancet Oncology*.

[8] Turner S., Chia S., Kanakamedala H. (2021). Effectiveness of alpelisib + fulvestrant compared with real-world standard treatment among patients with HR+, HER2–, *PIK3CA* -mutated breast cancer. *The Oncologist*.

[9] Cook M. M., Al R. L., Kaempf A. J., Saraceni M. M., Savin M. A., Mitri Z. I. (2021). Everolimus plus exemestane treatment in patients with metastatic hormone receptor-positive breast cancer previously treated with cdk4/6 inhibitor therapy. *The Oncologist*.

[10] Dhakal A., Antony Thomas R., Levine E. G. (2020). Outcome of everolimus-based therapy in hormone-receptor-positive metastatic breast cancer patients after progression on palbociclib. *Breast Cancer: Basic and Clinical Research*.

[11] Zhou J., Wu X., Zhang H. (2022). Clinical outcomes of tucidinostat-based therapy after prior CDK4/6 inhibitor progression in hormone receptor-positive heavily pretreated metastatic breast cancer. *The Breast*.

[12] Rugo H. S., Bardia A., Marmé F. (2023). Overall survival with sacituzumab govitecan in hormone receptor-positive and human epidermal growth factor receptor 2-negative metastatic breast cancer (TROPiCS-02): a randomised, open-label, multicentre, phase 3 trial [published online ahead of print, 2023 Aug 23]. *Lancet*.

[13] Li G. F., Qiao Y. W., Guan A. J., Yu G. (2023). Informative censoring of progression-free survival data in the TROPiCS-02 trial: an unrecognized bias. *Journal of Clinical Oncology*.

[14] Li Y., Li W., Gong C. (2021). A multicenter analysis of treatment patterns and clinical outcomes of subsequent therapies after progression on palbociclib in HR+/HER2− metastatic breast cancer. *Therapeutic Advances in Medical Oncology*.

[15] Shen G., Zheng F., Ren D. (2018). Anlotinib: a novel multi-targeting tyrosine kinase inhibitor in clinical development. *Journal of Hematology & Oncology*.

[16] Qian Y., Lou K., Zhou H., Zhang L., Yuan Y. (2022). Efficacy and safety of anlotinib-based treatment in metastatic breast cancer patients. *Frontiers in Oncology*.

[17] Syed Y. Y. (2018). Anlotinib: First global approval. *Drugs*.

[18] Sun Y., Niu W., Du F. (2016). Safety, pharmacokinetics, and antitumor properties of anlotinib, an oral multi-target tyrosine kinase inhibitor, in patients with advanced refractory solid tumors. *Journal of Hematology & Oncology*.

[19] Xi J., Oza A., Thomas S. (2019). Retrospective analysis of treatment patterns and effectiveness of palbociclib and subsequent regimens in metastatic breast cancer. *Journal of the National Comprehensive Cancer Network*.

[20] Das A., Mahapatra S., Bandyopadhyay D. (2021). Bleeding with vascular endothelial growth factor tyrosine kinase inhibitor: a network meta-analysis. *Critical Reviews in Oncology/Hematology*.

